# Development and validation of a clinical tool to semi-automatic measure three-dimensional TAR alignment on two-dimensional radiographs

**DOI:** 10.1186/s13047-023-00640-0

**Published:** 2023-06-24

**Authors:** Sanne W. G. van Hoogstraten, Joris Hermus, Vera Verbiest, Bert van Rietbergen, Jacobus J. C. Arts

**Affiliations:** 1 Department of Orthopedic Surgery, Laboratory for Experimental Orthopedics, Maastricht University Medical Center, Maastricht, the Netherlands; 2 Department of Biomedical Engineering, Orthopedic Biomechanics, Eindhoven University of Technology, Eindhoven, the Netherlands

**Keywords:** Total Ankle Replacement, Malalignment, Radiographic measurements, Subsidence, Migration

## Abstract

**Background:**

Malalignment is often postulated as an important reason for the high failure rate of total ankle replacements (TARs). The correlation between TAR malalignment and clinical outcome, however, is not fully understood. Improving and expanding radiographic TAR alignment measurements in the clinic might lead to a better insight into the correlation between malalignment and the clinical outcome. This study aims to develop and validate a tool to semi-automatic measure TAR alignment, and to improve alignment measurements on radiographs in the clinic.

**Methods:**

A tool to semi-automatically measure TAR alignment on anteroposterior and lateral radiographs was developed in MATLAB. Using the principle of edge contouring and the perpendicular relationship between the anteroposterior and lateral radiographs, the exact configuration of the TAR components can be found. Two observers validated the tool by measuring TAR alignment of ten patients using the tool. The Intraclass Coefficient (ICC) was calculated to assess the reliability of the developed method. The results obtained by the tool were compared to clinical results during radiographic follow-up in the past, and the accuracy of both methods was calculated using three-dimensional CT data.

**Results:**

The tool showed an accuracy of 76% compared to 71% for the method used during follow-up. ICC values were 0.94 (*p* < 0.01) and higher for both inter-and intra-observer reliability.

**Conclusions:**

The tool presents a reproducible method to measure TAR alignment parameters. Three-dimensional alignment parameters are obtained from two-dimensional radiographs, and as the tool can be applied to most TAR designs, it offers a valuable addition in the clinic and for research purposes.

## Background

The Total Ankle Replacement (TAR) has been increasingly used over the past years, as it has been shown to relieve pain while maintaining ankle function [1]. Despite the development of four generations of TAR designs, primary concerns regarding revision rates remain present. Studies reporting on the short-term outcome of TAR 3 years after surgery report a reoperation rate of up to 36%. After 14 years, only 46% of the TARs did not undergo revision surgery or conversion to arthrodesisand this high revision rate is of major concern [2–6]. Malalignment is an important predictor for the long-term outcome of TAR, and a very steep learning curve for TAR surgery is presentas the functional outcome of the TAR increases significantly with increasing surgical experience [7–9]. Also, correcting pre-operative deformity of the ankle is extremely challenging, and proper alignment is the key to a successful TAR surgery [10–13].

After TAR surgery, patients undergo follow-up appointments regularly to monitor TAR performance. During standard follow-up, TAR alignment is measured on an anteroposterior (AP) and lateral radiographs [14, 15]. Even though malalignment is often postulated as the main reason for TAR failure, a standard, and accurate TAR alignment measurement method lacks and measurable parameters on radiographs are limited. Only a few studies investigated the correlation between TAR alignment and clinical outcome, but different measurement methods were used, and varying output parameters were investigated [16–18]. No significant correlations between several TAR alignment parameters and the clinical outcome were found. Due to the inconsistency in methodology, comparison between the studies is difficult, and more importantly, the different TAR alignment measurement methods come with varying accuracy [19–21]. Furthermore, only little is known about TAR malalignment in the transverse plane, including relative axial alignment of the tibial and talar components. A study reported large inter-individual variability of axial rotation of the TAR components, which may impose an unknown contribution to TAR failure due to malalignment [22]. To gain more insight into the correlation between TAR malalignment and failure, large-scale measurements using a consistent and accurate method that is applicable in the clinic, allowing TAR alignment measurement on all rotational axes, are necessary.

In a previous study by Kitzen et al. (2020), a tool to measure three-dimensional (3D) parameters of a total disk replacement on two-dimensional (2D) spinal radiographs was developed [23, 24]. 3D alignment information of the total disk replacement could be obtained and correlated to clinical outcomes. Such a tool, when adjusted to TAR application, would be valuable in the clinic as it would expand the set of measurable TAR alignment parameters on plain radiographs, as 3D alignment parameters would be available without the use of a CT scan, saving health care costs and reducing the radiation dose. Radiographic TAR alignment measurements are always restricted to measuring parameters in the coronal and sagittal plane, while rotation in the transverse plane might as well be an interesting parameter. Such a semi-automated tool is expected to offer a more accurate method to measure TAR alignment. Therefore, the goal of this study was to develop a clinical tool to semi-automatic measure TAR alignment on radiographs, using the framework developed by Kitzen et al. (2020) and to validate the results relative to measurements based on CT scans that are taken as the gold standard. We compared the accuracy of this method compared to the alignment measurements that were performed using a standard manual method during follow-up. This tool is expected to expand TAR alignment measurements using radiographs and standardize research on correlating TAR malalignment to clinical outcomes. This will lead to a better insight into the role of malalignment in the failure mechanism of the TAR.

## Methods

### Patient selection

**Fig. 1:**
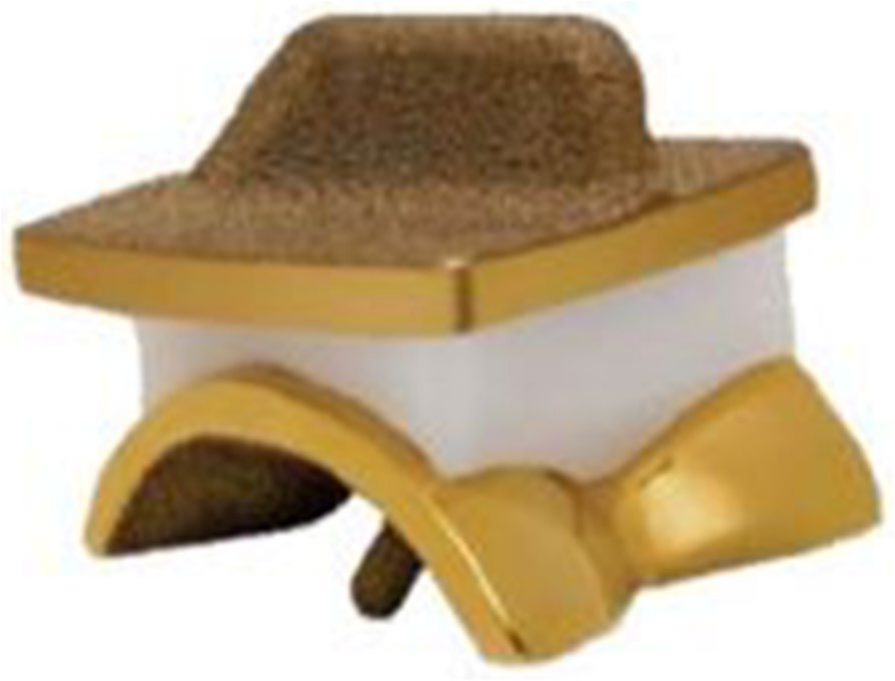
The CCI Evolution TAR implant (Van Straten Medical). The three components from top to bottom: tibial CoCrMo component, polyethylene liner, talar CoCrMo component

An anonymized cohort consisting of 61 patients that received the CCI Evolution TAR (Fig. 1) cohort at Maastricht University Medical Center was reviewed, and cohort demographics are shown in Table 1. To test the accuracy and reliability of the tool, specific data needed to be available, and the patients meeting the following criteria were selected from the cohort:

1. A postoperative computed tomography (CT) scan was present
2. An anteroposterior and lateral weight-bearing radiograph obtained within a week from this CT scan was present
3. TAR alignment of the patient was measured during follow-up, and measurement outcomes were registered in the database. In this database, coronal and sagittal alignment of the tibial component and coronal alignment of the talar component was registered.

**Table 1:** Exclusion criteria and characteristics of included patients from the CCI Evolution TAR cohort at MUMC

**Total number of patients in CCI cohort**	61
**Number of patients after applying the following exclusion criteria:**	
1. Follow-up data including manual measurement results available	59
2. Number of patients with post-operative CT scan available	18
3. Radiographs corresponding to post-op CT scan available	10
**Characteristics of study population**	
Study size	10/10 ankles/subjects
Gender	7/3 male/female
Mean age at operation	68.6 ± 4.2 years
Operation side	3/7 right/left
Operation date	23/04/2010—16/03/2016
Mean follow-up	57.6 ± 33.1 months

Ten patients fulfilled these criteria and were included in this study.

### Tool development and usage

**Fig. 2:**
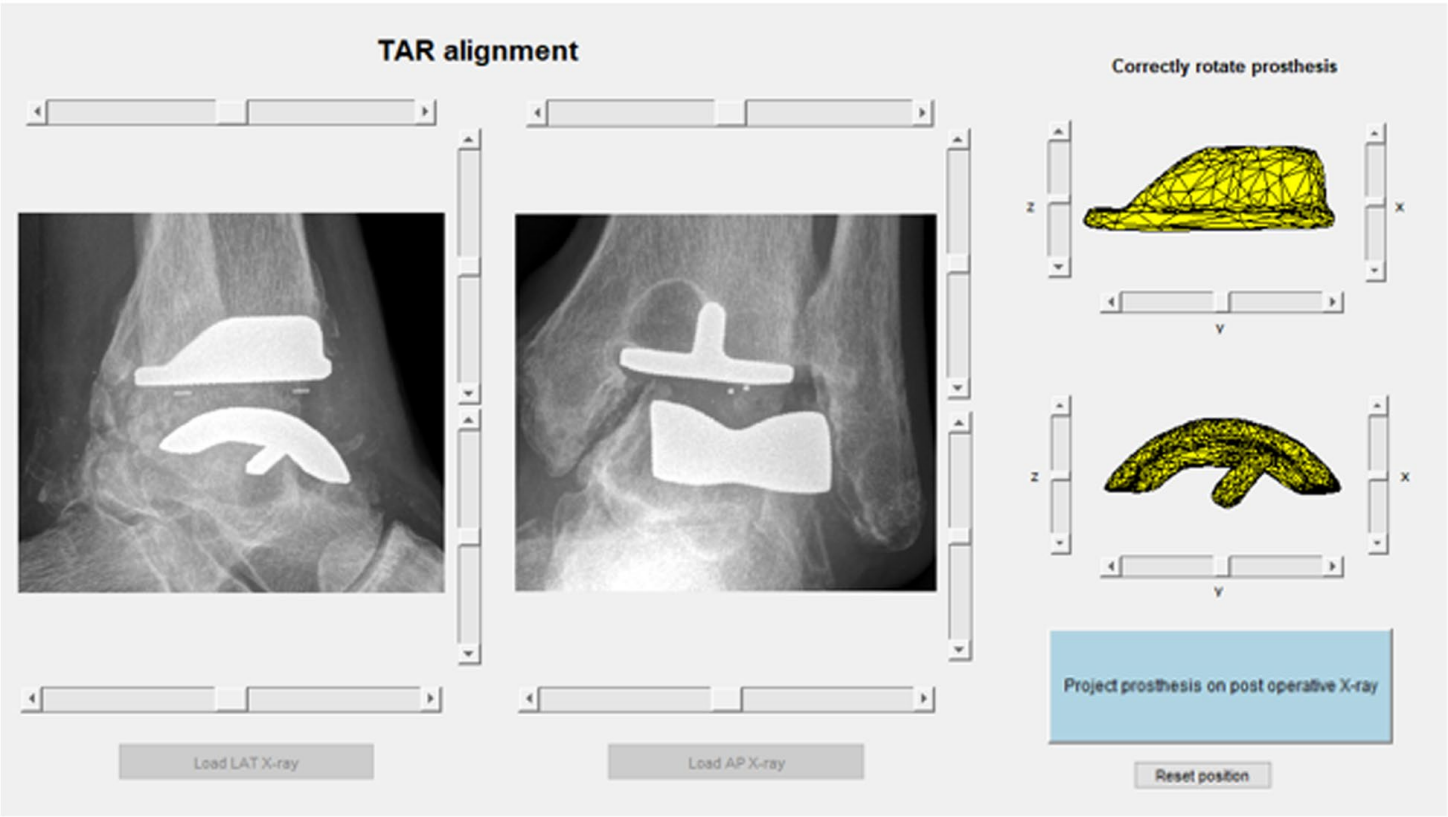
The graphical user interface of the tool displaying the lateral and anteroposterior radiographs (on the left) and 3D reconstructions of the tibial and talar CCI Evolution TAR component (on the right). Sliders around the radiographs to translate the contours of the 3D CCI parts, sliders around the 3D reconstructions to rotate the contours of the 3D CCI parts

3D reconstructions of the standard-sized tibial and talar components of the CCI Evolution TAR design were obtained by 3D scanning the individual components using the ATOS Scanport (Zebicon a/s; Billund). Using a custom-developed software package implemented in MATLAB (MATLAB; R2019b, MathWorks, Natick, MA, USA), these 3D reconstructions in neutral alignment were visualized in a graphical user interface (GUI). Secondly, a custom-developed software package based on the work of Kitzen et al., (2020) [24] was implemented in MATLAB, to simultaneously display both lateral and AP radiographs in the same GUI (Fig. 2).

**Fig. 3:**
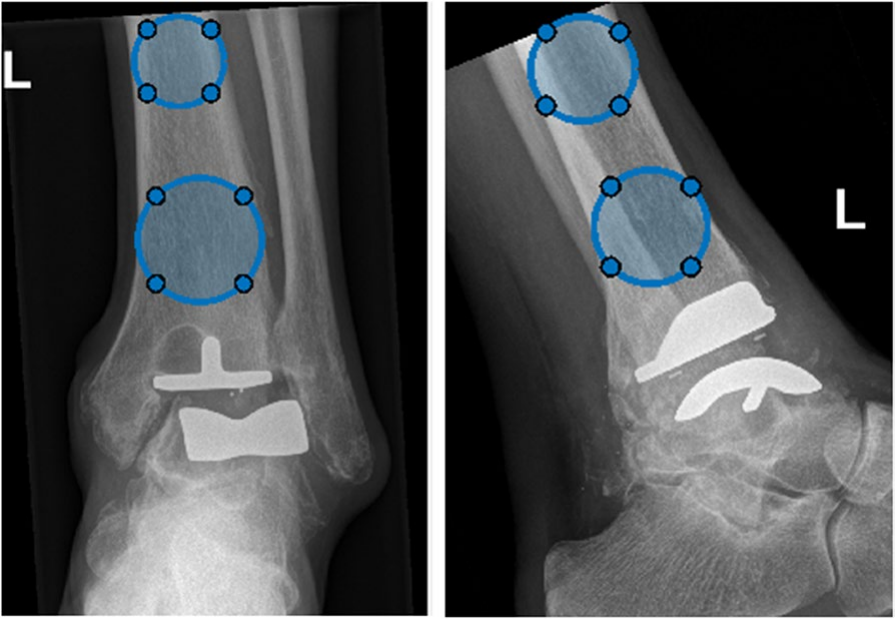
Circles fitted on the tibial shaft of the tibia to measure the anatomical tibia axis

After the radiographs are loaded in the GUI, the user is asked to select the tibial axis by aligning two circles on the tibial shaft, one most proximally and one most distally (Fig. 3). The tibial axis will serve as a reference line for rotation measurements in the weight-bearing radiographs. After selecting the tibial axis, images are cropped around the TAR for visualization purposes during the contouring process.

**Fig. 4:**
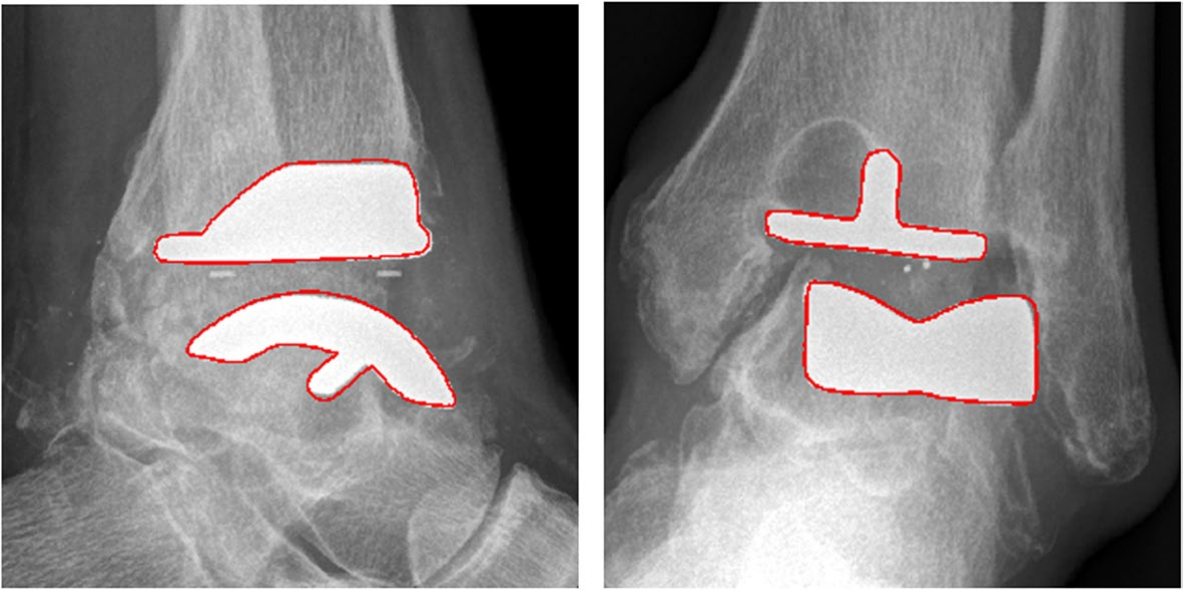
Contour mapping of the 3D TAR component contours on the lateral and anteroposterior radiographs

After selecting the tibial axis, the contours of the 3D-scanned TAR components are plotted on top of the radiographs. The user can translate and rotate the contours of the 3D TAR components, using the sliders next to respectively the radiographs and 3D TAR components (Fig. 2), until the contours of the 3D components match the contours of the TAR on the radiographs. The radiographs depicted in Fig. 2 are cropped around the TAR for better visualization, but the tibial axis measured before cropping is saved. When rotating the 3D components, contours on both radiographs rotate simultaneously due to the perpendicular relationship of the anteroposterior and lateral radiographs. This way, only one configuration of the 3D scanned TAR components can be found to fit the contours on both radiographs simultaneously (Fig. 4).

### Radiological analysis

Once the 3D components are rotated such that the contours fit the TAR contours on both radiographs (Fig. 4), TAR alignment parameters are obtained. TAR component rotation along the x, y, and z-axis and distance between the TAR components along the x- and y-axis is calculated from the corresponding slider values. The x-axis is equal to the tibial axis, used as a reference line to map the TAR contours on the radiographs to find the relative TAR component position. A MATLAB script was developed to save all measured data automatically in a database, together with clinical patient data. Differences in TAR alignment are automatically detected when new data of the same patient is saved to the database.

### Reliability and statistics

To determine the inter-observer reliability of the tool, two observers measured TAR alignment on the radiographs of the selected patients using the developed tool. One observer performed a second measurement on the same radiograph six weeks after the first measurement, to determine the intra-observer reliability. The means of the measured outcomes were used in further statistical analysis. Using IBM SPSS 22.0,

Intra-class Correlation Coefficients (ICC) were calculated to determine inter-and intra-observer reliability [25]. TAR rotation along the x, y, and z-axis was included in the results, as well as the distance between the centers of the contours of the 3D components in the lateral-medial and anterior–posterior direction. The outcome measures of the manual method used in clinical practice were compared to the outcomes obtained with the semi-automatic tool, using paired t-tests with a significance level of *p* < 0.05.

### Accuracy

**Fig. 5:**
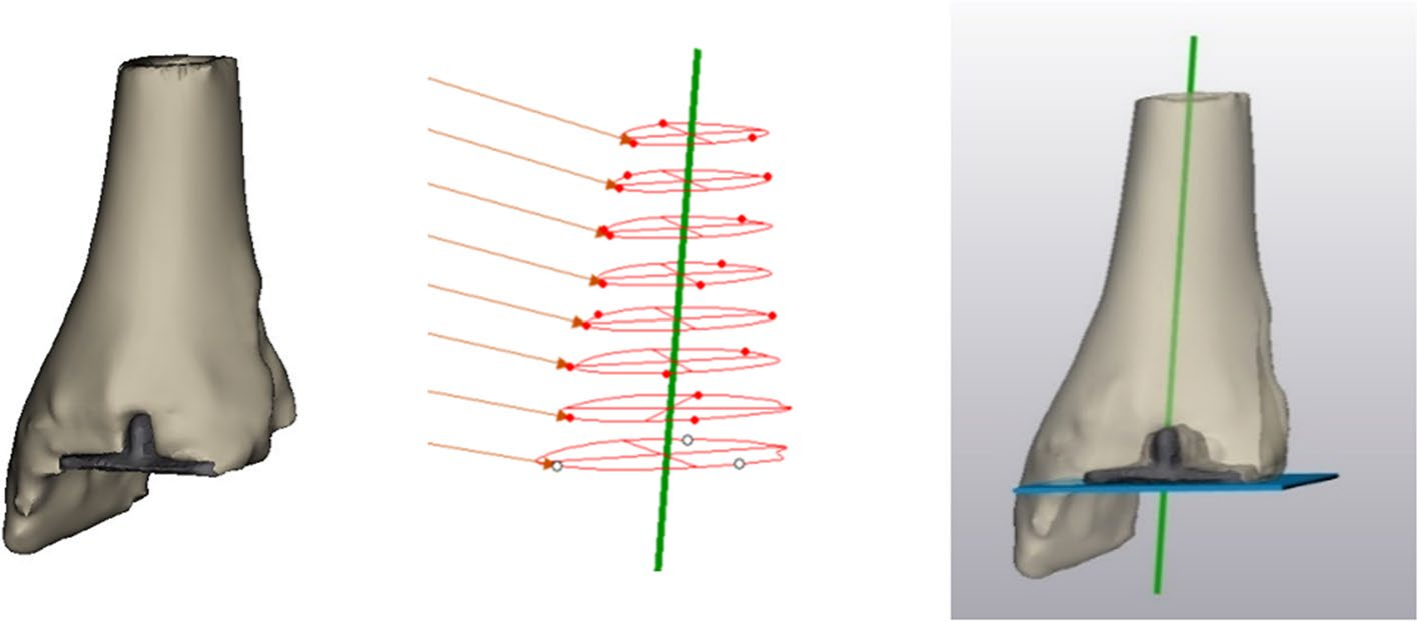
Tibial axis measurement in a 3D reconstruction, by fitting a line through centroids of the tibial shaft

The available postoperative CT scans of the ten selected patients in this study were used to test the accuracy of the tool. Coronal and sagittal alignment—corresponding to rotation along the x- and y-axis in the tool, respectively – were calculated in a 3D reconstruction of the tibias with tibial TAR components. The 3D reconstructions were created by segmenting CT scans in Mimics (Research 22.0; Materialise NV, Leuven, Belgium). TAR alignment was measured by obtaining the angle between the tibial axis and the plateau of the tibial TAR component. The tibial axis was obtained by finding the centroids along the tibial shaft and fitting a line through these centroids (Fig. 5) [26]. The tibial plateau was obtained by fitting a line along the bottom plateau of the tibial TAR component. The angle between the tibial axis and tibial TAR in the coronal and sagittal plane is regarded as the true TAR alignment. The percentage of accuracy was calculated using $\%\;accuracy=(1-abs\left(\frac{{V}_{A}-{V}_{O}}{{V}_{A}}\right))*100\%$ with ${V}_{A}$ being the accepted value obtained from the 3D reconstruction, and ${V}_{O}$ being the outcome obtained from the semi-automatic tool.

As the database of the manual TAR alignment measurements obtained during the follow-up of the ten patients was present as well, the accuracy of this manual method was calculated and compared to that of the newly developed semi-automatic approach.

## Results

### Reliability

Results for the rotation and component distance measurements are given in respectively Tables 2 and 3. The average differences of the measured angles and distances between the two observers were less than 1 degree and 0.4 mm, respectively. No significant differences were found. Two patients were excluded due to a mismatch in foot position between the anteroposterior and lateral radiograph, of which the effect will be discussed later. For both the rotation and component distance measurement results, high correlation coefficients between the two observers were found with an ICC ≥ 0.80. Also, high intra-observer correlation coefficients were found for all measurements with an ICC ≥ 0.95 (Tables 2 and 3).

**Table 2:** Tibial and talar rotation in the coronal and sagittal plane per observer (in degrees), given as mean with standard deviation (SD) with absolute difference between observers (∆), and inter-observer results with intraclass correlation coefficient and *p*-value between both observers (*n* = 8)

	Tibial alignment (°)		Talar alignment (°)	
**Observer**	**Coronal** Mean ± SD	**Sagittal** Mean ± SD	**Coronal** Mean ± SD	**Sagittal** Mean ± SD
1	5.8 ± 3.6	4.1 ± 2.8	4.8 ± 3.5	4.6 ± 2.7
2	6.4 ± 3.2	3.9 ± 3.1	4.0 ± 3.2	5.4 ± 2.8
∆	0.6	0.2	0.8	0.8
**ICC (*p*-value)**				
Means 1–2	0.98 (*p* < 0.01)	0.98 (*p* < 0.01)	0.80 (*p* < 0.01)	0.95 (*p* < 0.01)
Means 1a-1b	0.98 (*p* < 0.01)	0.98 (*p* < 0.01)	0.98 (*p* < 0.01)	0.96 (*p* < 0.01)

**Table 3:** The distance between the tibial and talar component (in mm) over the x- and y-axis, given as mean with standard deviation (SD) with absolute difference between observers (∆), and inter-observer results with intraclass correlation coefficient and *p*-value between both observers (*n* = 8)

	Distance between the tibial and talar component along the x-axis (mm)		Distance between the tibial and talar component along the y-axis (mm)	
**Observer**	**Medial–lateral** Mean ± SD	**Anterior–posterior** Mean ± SD	**Medial–lateral** Mean ± SD	**Anterior–posterior** Mean ± SD
1	2.4 ± 1.5	5.9 ± 2.2	18.1 ± 2.2	19.0 ± 1.2
2	2.3 ± 1.7	6.1 ± 2.4	18.5 ± 1.7	18.9 ± 1.6
∆	0.1	0.2	0.4	0.1
**ICC (*p*-value)**				
Means 1–2	0.95 (*p* < 0.01)	0.98 (*p* < 0.01)	0.90 (*p* < 0.01)	0.93 (*p* < 0.01)
Means 1a-1b	0.92 (*p* < 0.01)	0.96 (*p* < 0.01)	0.92 (*p* < 0.01)	0.93 (*p* < 0.01)

### Manual measurement method vs. semi-automatic tool

Mean outcome parameters obtained by the manual measurement method and the semi-automatic tool are reported in Table 4. Results from the paired t-tests show no significant differences between the two measurement methods, probably due to the small sample size.

**Table 4:** Mean results for tibial and talar rotation (in degrees) in the coronal and sagittal plane, given as mean value per measurement with absolute difference between methods (∆), including the *p*-value to compare means of both measurement methods (*n* = 8)

	Tibial alignment (°)		Talar alignment (°)
	**Coronal** Mean ± SD	**Sagittal** Mean ± SD	**Coronal** Mean ± SD
Manual	4.6 ± 3.4	3.5 ± 2.7	4.3 ± 3.7
Semi-automatic	6.1 ± 3.4	4.0 ± 2.8	4.4 ± 3.3
∆	1.5	0.5	0.1
*p*-value	*p* > 0.05	*p* > 0.05	*p* > 0.05

### Accuracy

For the eight patients included in the results, 3D reconstructions of the tibia with tibial components were made and alignment of the tibial component was measured. The accuracy of both the presented tool and the manual measurement method was calculated. The average accuracy was 77% and 70% for the semi-automatic and manual methods, respectively (Table 5).

**Table 5:** Accuracy of the semi-automatic and manual measurement methods. Mean accuracy of the coronal and sagittal alignment of the tibia component of all patients is reported

Measurement method	Accuracy of coronal tibial alignment measurements	Accuracy of sagittal tibial alignment measurements
Semi-automatic	81%	73%
Manual	68%	71%

## Discussion

The purpose of this study was to develop and validate a semi-automatic clinical tool to measure TAR alignment on plain radiographs. TAR alignment of eight patients from the CCI Evolution cohort was measured using the tool, and accuracy and reliability were calculated. By providing this semi-automatic tool, which standardizes TAR alignment measurements and expands the set of measurable parameters using radiographs, research on the correlation between TAR alignment, migration, and clinical outcome will be improved and facilitated. With this technique, this can be reached without additional radiation doses or costs as no CT scan is necessary to find the 3D alignment of the TAR components. Relative migration of the TAR components can be detected by applying this tool on radiographs of the same patients at different time points during follow-up. High inter-and intra-observer reliability was found (ICC ≥ 0.94), so the tool is a reproducible and reliable method for measuring TAR alignment. On average, the accuracy of the coronal alignment measurements increased by 13% and the accuracy of the sagittal alignment measurement showed a small increase of 2% when using the tool compared to the manual method. Now that the tool is validated, future studies must be performed to apply the tool to the CCI cohort in order to gain insight into the relationship between TAR malalignment and the clinical outcome. The tool presented in this study is applicable to any TAR design as long as one asymmetric plane is present in the TAR components, which for most TAR designs is true.

When implemented in the clinic, the tool also standardizes the tibial axis measurements. The tibial axis measurement method that was implemented resulted from descriptions found in the literature [27–32], and was based on a previous study investigating the most accurate method to measure the longitudinal tibial axis [21]. In this study, the anatomical axis was selected, but the tool allows the user to choose the mechanical axis, or any other axis if preferred, as long as the user is consistent in using the same axes during the follow-up of a patient. With the manual TAR alignment measurement method, the tibial axis is drawn by hand. Although it must be confirmed by future studies, it is expected that the accuracy and reliability of the anatomical tibial axis measurements will increase upon implementation of the tool. What must also be pointed out regarding the tibial axis measurements, is that it is essential to obtain lower-leg radiographs during clinical follow-up, as too distally tibial radiographs can give inaccurate results when measuring the tibial axis [27]. In the current study, some radiographs only showed a distal part of the tibia. Still, the tool showed higher accuracy than the manual measurement method, when compared to CT data. This shows that upon the usage of full-leg images, the tool will show even higher accuracy when compared to the CT data. With future use of the developed tool, it is recommended that at least lower-leg radiographs are used. However, it must be stated that in clinical practice optimal radiograph quality cannot always be guaranteed. Even in a suboptimal situation, this tool shows that more accurate TAR alignment measurements can be obtained.

Other methods to assess TAR position and kinematics have been frequently applied in TAR research, such as in vivo 3D fluoroscopy and radiostereometric analysis (RSA). These methods give detailed insight into the kinematic behavior of TAR components [33–39]. However, these techniques require a prospective study design, whereas the presented tool in this research can be applied retrospectively to study TAR component position, using radiographs obtained during routine follow-up consults. This shows the main strength of the presented tool, as it serves as an alternative measurement method, standardizing and improving the accuracy of TAR measurements, which can be used to study TAR failure in a retrospective manner. Compared to 3D fluoroscopy and RSA, this makes the presented method a valuable tool in TAR research without additional impact on the patient or increase in healthcare costs.

Some limitations of the current study must be discussed. Firstly, we assumed that the lateral and anteroposterior radiographs were perpendicular. However, since radiographs were obtained during regular patient care without a reference line or bilateral imaging, it is possible that the anteroposterior and lateral radiographs were not exactly perpendicular. Large discrepancies, however, would have been detected as it would make it impossible to find the configuration of the 3D TAR components. However, the presence of perpendicular radiographs is crucial for the tool to add value in the clinic. A problem regarding radiograph quality was encountered during measurements of two of the ten selected patients, which showed a moving artifact in between obtaining the anteroposterior and lateral radiograph. Since the tool relies on the perpendicularity and coupling of these two radiographs, foot movement leads to the fact that it is impossible to find a matching configuration of the TAR components. This major problem, however, can be resolved in the clinic by easy fixation of the foot using a brace or holder for example. More importantly, when foot fixation is guaranteed using a brace, the tool presents a method to measure sagittal talar alignment, which currently does not exist yet, and also for this output parameter high accuracy and reliability are expected. Furthermore, this would allow the addition of a landmark on both the lateral and anteroposterior radiograph, expanding the TAR position measurements to absolute values as well. Weight-bearing CT imaging would be a valuable addition for the validation of our tool and using WBCT will take away problems regarding foot movement in plain radiography [40, 41]. Unfortunately, WBCT was not available at the moment in our clinical center. Another limitation is that only inter and intra-observer reproducibility was tested. A more thorough reproducibility would require making multiple radiographs of the same patient with more observers. Furthermore, the sample size of this study was small, and validating the tool with a larger sample size will increase the robustness of the validation. However, regarding the convincing ICC values and the fact that validity was determined comparing to CT data as the golden standard, the small sample size and the fact that intra-observer reliability was only obtained for one observer study were not considered as major limitations. However, a larger scale study is expected to show convincing results on the accuracy and reliability of the presented tool. Also, statistical analysis was limited due to the small sample size and the continuous outcome parameters. Besides, the selection criteria that were applied to the cohort that led to the small sample size, were chosen for validation purposes as the presence of CT data was required. Furthermore, the 3D reconstructions to calculate accuracy were developed according to a method presented in the literature showing high intraclass agreement [26]. However, the accuracy of the talar component alignment measurements was not calculated, since the foot is positioned differently during a CT scan compared to weight-bearing radiographs and the tibial axis cannot be used as a reference line. After validation, the tool can be broadly applied to patients with a TAR as CT data will not be necessary for application of the tool. Applying the tool to a larger dataset to gain clinical information was, however, outside of the scope of this study. Also, the current tool is not fully automated but in future development, this could be reached by using automatic edge-detection algorithms [42–45]. This will result in a very efficient method to measure TAR alignment, which will greatly enhance insight into the relationship between TAR alignment and clinical outcomes. The accuracy values found when comparing the radiographic TAR measurement methods to measurements using a CT scan, show the importance of improving these radiographic TAR alignment measurements. Lastly, in this study, the tool was used to measure TAR alignment during follow-up, but upon further development the tool has potential as a pre-operative mapping tool, finding the optimal TAR alignment per patient.

## Conclusions

Concluding, a reproducible tool was developed to semi-automatically measure TAR alignment on anteroposterior and lateral radiographs. Only tibial alignment measurements were included to validate the tool, as the talar component shifts relative to the tibial component from weight-bearing radiographs to non-weight-bearing CT scans. Future research should be dedicated to the automation and application of the tool on a larger dataset. The developed tool increases accuracy from the current measurement method but more importantly allows measurement of 3D alignment parameters on 2D radiographs, increasing insight into TAR position. The tool facilitates research on the relationship between TAR alignment, migration, and clinical outcome, by standardizing and expanding TAR alignment measurements.

## Data Availability

All data generated and analyzed during this study are included in this published article or depicted in figures in this published article.
